# Current evidence on drug therapy for dementia in people with Down syndrome: an overview of systematic reviews

**DOI:** 10.1590/1980-5764-DN-2024-0241

**Published:** 2025-09-01

**Authors:** Ticiano Gomes do Nascimento, Ana Clara Lira do Nascimento, Maria Abath Campos, Raphaella Amanda Maria Leite Fernandes

**Affiliations:** 1 Universidade de Pernambuco, Curso de Medicina, Garanhuns PE, Brazil.; 2 Universidade de Pernambuco, Faculdade de Ciências Médicas, Curso de Medicina, Recife PE, Brazil.; 3 Universidade Federal de Pernambuco, Faculdade de Ciências Médicas, Núcleo de Ciências da Vida, Recife PE, Brazil.

**Keywords:** Down Syndrome, Dementia, Drug Therapy, Systematic Review, Síndrome de Down, Demência, Quimioterapia, Revisão Sistemática

## Abstract

Individuals with Down syndrome (DS) are at a higher risk of developing dementia at an earlier age. Identifying and summarizing studies that provide strong evidence in drug therapy is crucial for aiding healthcare professionals in clinical decision-making.

**Objective:**

To identify systematic reviews (SRs) published in various databases and evaluate the effectiveness of pharmacological interventions for treating dementia specifically in individuals with DS.

**Methods:**

SRs published in the United States National Library of Medicine (PubMed), Scopus, Web of Science, Biblioteca Virtual em Saúde (BVS), and Cochrane Library over the past ten years were included. The methodological quality of the SRs was assessed using the AMSTAR-2 and the Grading of Recommendations Assessment, Development and Evaluation (GRADE) tools.

**Results:**

Five SRs were included, analyzing the effects of simvastatin, antioxidants, acetyl-L-carnitine, anticonvulsants, acetylcholinesterase inhibitors (donepezil), NMDA receptor antagonists (memantine), and fast-acting intranasal insulin on dementia symptoms in DS patients.

**Conclusion:**

All SRs indicated low — or less — certainty of evidence, suggesting that various pharmacological approaches should be interpreted with caution.

## RESUMO

Indivíduos com síndrome de Down (SD) apresentam maior risco de desenvolver demência em idade mais precoce. Identificar e resumir estudos que forneçam evidências sólidas em terapia medicamentosa é crucial para ajudar os profissionais de saúde na tomada de decisões clínicas.

**Objetivo:**

Identificar revisões sistemáticas (RS) publicadas em diversas bases de dados e avaliar a eficácia das intervenções farmacológicas no tratamento da demência especificamente em indivíduos com SD.

**Métodos:**

Foram incluídas revisões sistemáticas publicadas na Biblioteca Nacional de Medicina dos Estados Unidos (PubMed), Scopus, Web of Science, Biblioteca Virtual em Saúde (BVS) e Cochrane Library nos últimos dez anos. A qualidade metodológica das RS foi avaliada utilizando as ferramentas AMSTAR-2 e Grading of Recommendations Assessment, Development and Evaluation (GRADE).

**Resultados:**

Cinco revisões sistemáticas foram incluídas, analisando os efeitos de sinvastatina, antioxidantes, acetil-L-carnitina, anticonvulsivantes, inibidores da acetilcolinesterase (donepezil), antagonistas do receptor NMDA (memantina) e insulina intranasal de ação rápida nos sintomas de demência em pacientes com SD.

**Conclusão:**

Todas as revisões sistemáticas indicaram baixa ou menor certeza das evidências, sugerindo que as diversas abordagens farmacológicas devem ser interpretadas com cautela.

## INTRODUCTION

Down syndrome (DS) is the most prevalent chromosomal anomaly at birth, resulting from the presence of a third copy of chromosome 21 or a translocation involving this chromosome. These genetic alterations determine specific characteristics of the syndrome, including learning difficulties, craniofacial modifications, and decreased muscle tone in early childhood^1^. Advances in health care and environmental conditions for individuals with DS have significantly improved their life expectancy, which in developed countries can now reach an average of 58 years. However, this is still approximately 20 years shorter than the life expectancy of individuals without trisomy^2^.

As individuals with DS live longer, they face a higher prevalence of age-related health conditions, including dementia, which significantly impacts both quality of life and mortality. Dementia is a syndrome that is related to various cognitive factors of the individual, such as memory, reasoning, and the ability to perform daily activities. In people that live with DS, this condition may arise from multiple causes, including Alzheimer’s disease (AD) and other pathological mechanisms^3^. The increased risk of early-onset dementia in this population is linked to neuroanatomical and biological changes associated with DS. Specifically, the presence of a third copy of chromosome 21 leads to the overexpression of genes such as *APP* and *DYRK1A*, which play a critical role in the production and deposition of beta-amyloid (Aβ) protein and neurofibrillary tangles—hallmarks of AD pathology. Furthermore, neuroinflammation and oxidative stress contribute to the neuropathological changes associated with cognitive decline. In addition, individuals with DS exhibit additional brain abnormalities, including reduced brain size, alterations in gray and white matter, and compromised cortical development^4^.

The high prevalence of dementia and its associated mortality risk among individuals with DS is a pressing concern. Studies have shown that nearly all individuals with DS exhibit neuropathological features of AD by their fourth decade of life, with dementia diagnoses becoming increasingly common with age. A 20-year longitudinal study reported that 97.4% of individuals with DS aged 35 years or older developed dementia, with most diagnoses occurring before the age of 55^5^. Dementia not only reduces functionality and independence but also significantly impacts mortality rates, as highlighted in recent studies^6^.

Currently, the pharmacological treatment options for cognitive decline and dementia in individuals with DS remain limited and inconsistent. While cognitive decline in DS is frequently linked to AD pathology, it may also result from other mechanisms, highlighting the need for a broader investigation of therapeutic approaches. The existing literature suggests disparities in treatment efficacy between the general population and individuals with DS, further emphasizing the necessity for targeted research and clinical trials^3^.

This overview aims to address the challenges and gaps in the current understanding and treatment of cognitive decline in individuals with DS, focusing on both AD-related and non-AD-related causes. By exploring the available evidence, this review seeks to provide insights into potential therapeutic approaches and emphasize the importance of further research to improve outcomes for individuals with DS experiencing cognitive decline.

### Rationale

As research in the field progresses, many reviews addressing the use of therapeutic drugs for the treatment of dementia in individuals with DS have been published over the years. These reviews are found in various databases and summarize the evidence individually, evaluating a specific therapeutic drug within the broad range of interventions available for managing dementia in the context of DS. However, a succinct summary of the findings from these reviews across different types of treatments has not been conducted. An overview of the systematic reviews is deemed appropriate to summarize the evidence across the spectrum of interventions^7^.

The primary objective of this overview of reviews is to synthesize current evidence and evaluate the efficacy of pharmacological interventions for cognitive decline in individuals with DS, with specific questions including:

- What is the current evidence regarding the efficacy of therapeutic drugs in improving cognitive function in this population?
- What gaps exist in research on the treatment of dementia in individuals with DS?
- Which cognitive domains are most affected by the interventions?

## METHODS

This study followed the recommendations of the Preferred Reporting Items for Systematic Reviews and Meta-Analyses (PRISMA), a tool that presents a standardized approach for the development of reports, systematic reviews, and meta-analyses^8^.

### Inclusion and exclusion criteria

#### Study type

Systematic reviews and meta-analyses investigating the efficacy of pharmacological interventions for the treatment of dementia in people with DS were included. Narrative reviews, ongoing articles, conference proceedings, and qualitative studies were excluded.

#### Population

The target population included individuals diagnosed with DS, regardless of age or treatment context.

#### Intervention and comparison

The intervention considered was pharmacological treatment for different types of dementia, compared to placebo or usual treatment.

#### Outcome

The primary outcomes were improvements in cognitive and functional aspects, based on standardized assessments reported in the included studies.

### Search strategy and study selection

#### Research question formulation

The guiding question was constructed using the “population, intervention, comparison, and outcome” (PICO) strategy, resulting in the following question: “What is the current evidence of pharmacological therapy for treating dementia in people with DS?”. In this sense, the keywords contained in the question were located and converted to the most appropriate descriptors indexed in the Medical Subject Headings (MeSH) database. Finally, these terms were combined using the Boolean operators “AND” and “OR”, resulting in the search strategy.

#### Search strategy

The search strategy was developed using terms indexed in MeSH, combined with Boolean operators to enhance the scope and specificity of the search: (“Down Syndrome”) AND ((“Dementia”) OR (“Alzheimer Disease”) OR (“Frontotemporal Dementia”) OR (“Mixed Dementia”) OR (“Dementia, Vascular”) OR (“Lewy Body Disease”)) AND (“Drug Therapy”).

#### Data sources and search period

The searches were conducted in August 2024 in the following databases: United States National Library of Medicine (PubMed), Scopus, Web of Science, Biblioteca Virtual em Saúde (BVS), and Cochrane Library. Articles published between 2014 and 2024 in English, Portuguese, or Spanish were included.

### Study selection

#### Initial screening

The initial screening was conducted using Rayyan software, with two independent reviewers evaluating titles and abstracts for inclusion. Discrepancies were resolved by a third reviewer.

#### Full reading and data extraction

The selected articles were read in full, and data were extracted into a structured Excel spreadsheet. The information collected included the title, year of publication, authors, study type, country, population characteristics, age range, study objective, methodology, conclusions, outcomes, and risk of bias.

### Quality and risk of bias assessment

#### Tools used

The methodological quality of the included systematic reviews was assessed using the AMSTAR 2^9^. The AMSTAR-2 is a tool composed of 16 items:

- Research question and inclusion criteria according to the components of PICO (Population, Intervention, Comparators, Outcomes)
- Study planning protocol
- Justification for the selection of the study design for inclusion in the review
- Comprehensive literature search strategy
- Study selection in duplicate
- Data extraction in duplicate
- Report of excluded studies and justifications for exclusions
- Characteristics of the included studies described in adequate detail
- Methods to assess the risk of bias in the included studies
- Reporting of the funding sources of the included studies
- Methods for statistical combination of results (meta-analysis)
- Potential impact of the risk of bias in meta-analyses
- Consideration of the risk of bias in the interpretation and discussion of the results
- Discussion and explanation of heterogeneity
- Investigation of publication bias
- Conflict of interest report of the authors of the review

Each domain is classified as entirely suitable (“yes”), partially adequate (“partially yes”), not adequate (“no”) or not applicable (“n/a”). Some of these are considered critical. The assessment classifies the SRs according to the following degrees of confidence: critically low (more than one critical failure), low (a critical failure), moderate (more than one noncritical failure), and high (none or one noncritical failure). Furthermore, the quality of the evidence was classified using the GRADE appraisal tools method^10^.

### Evaluation criteria

GRADE considered five main items: risk of bias, inconsistency of results, indirectness of evidence, imprecision of results, and publication bias.

### Data synthesis

#### Analysis and interpretation of results

The study results were interpreted qualitatively, synthesizing the evidence into summary tables that included the risk of bias and quality of evidence assessment.

#### Discussion and conclusion

Discussion of the results was focused on interpreting the collected evidence, considering limitations, clinical implications, and areas for future research.

## RESULTS

### Study selection

**Figure 1:**
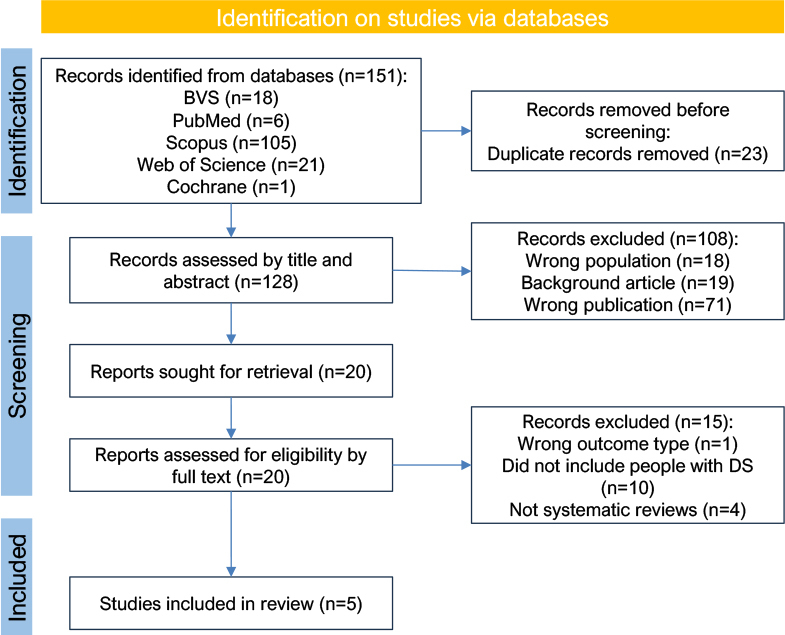
Preferred Reporting Items for Systematic Reviews and Meta-Analyses (PRISMA) flowchart of the study selection process

Source: Authors, 2024.

A total of 151 articles were found based on the descriptors: 105 articles in Scopus, 21 articles in the Web of Science, 18 in the BVS, six articles in PubMed, and one article in Cochrane Library. Before screening, 23 records were removed due to duplication. Then, 128 studies were assessed by title and abstract; 71 were the wrong publication type, 19 were background articles and 18 had the wrong population, resulting in 108 records excluded. Then, the other 20 were sought for retrieval. In the last stage of screening, 20 records were assessed for eligibility by full text, and 15 were excluded in total, ten due to having the wrong population (not including people with DS), four due to being the wrong publication type (not systematic reviews), and one due to having the wrong outcome. After applying the inclusion and exclusion factors, five articles were considered eligible for this review, as shown in the figure below (Figure 1).

### Characteristics of the studies

Table 1 ^11,12,13,14,15^, summarizes the characteristics of the included studies. Most of the included systematic reviews have a recent publication date, with four out of five reviews published in the last two years from the date of this overview’s search. Some studies evaluated multiple pharmacological interventions, but only the relevant data for the interventions included in this overview were extracted. The studies examined a variety of treatments, including simvastatin, antioxidant, acetyl-L-carnitine, anticonvulsants, acetylcholinesterase inhibitor (donepezil), NMDA receptor antagonists (memantine), and fast-acting intranasal insulin.

**Table 1:** Characteristics of included systematic reviews

Reference	Population criteria & age	Intervention	Comparison	Outcome	Quality of the Evidence (grade)
Wang et al.^11^	40 male participants.Range of age: 18–30 years.Mean age of participants: 20.2 years intervention, 21.5 years control.	L-Carnitine.	Placebo	No significant improvement in relevant indicators for DS patients.	Overall Neurological Efficacy: Low (due to inconsistency and imprecision).
Livingstone et al.^12^	Down’s Syndrome (DS) participants aged 18 and above. 427 participants.	Memantine, Simvastatin, Donepezil, Antioxidant, Acetyl-L-carnitine.	Placebo	Overall insufficient evidence to confirm effectiveness.	Cognitive Efficacy: Low (due to inconsistency and imprecision).
Oliveira e Faria^14^	Adults with DS with signs of dementia. 626 participants.	Antioxidant, acetylcholinesterase inhibitor (donepezil), NMDA receptor antagonists (memantine), fast-acting intranasal insulin.	Placebo.	The use of pharmacological therapies did not demonstrate relevant effects in the treatment of dementia.	Cognitive Efficacy: Low (due to inconsistency and imprecision).
Corniello et al.^15^	46 patients with DS, men and women.Progressive cognitive decline, Diagnosis of Alzheimer’s disease (AD) in all cases.	Levetiracetam (LEV) monotherapy, valproic acid (VPA) monotherapy and other anticonvulsants polytherapy.	No placebo group/usual care defined.	LEV and VPA showed great efficacy in seizure control. Treatment with LEV may enhance memory deficits, leading to amelioration of cognitive performances.	Evidence Certainty: Low (due to risk of bias, inconsistency and imprecision)
Islam et al.^13^	Down’s Syndrome (DS) participants aged 15 and above. 168 participants.	Memantine	Placebo	No significant effect on the outcomes of cognitive function.	Cognitive Efficacy: Low (due to risk of bias and imprecision)

The studies primarily focused on individuals with DS, aged over 15 years. The severity of cognitive decline among the participants was not uniformly reported but included adults showing signs of dementia, including those diagnosed with AD. Overall, the studies indicate that most pharmacological therapies did not show significant improvements in cognitive function or related outcomes, with several studies assessed as having critically low quality of evidence. The cognitive domains affected by the pharmacological interventions were also not uniformly reported, and most of the included reviews lacked sufficient studies to ensure the efficacy of the proposed interventions.

### Methodological quality

All included systematic reviews were assessed as low or critically low methodological quality on the AMSTAR-2 checklist. The only review that was assessed as low quality on the AMSTAR-2 checklist was the Cochrane systematic review^12^. It’s important to mention that the only factor that made the Cochrane review get assessed as low were insufficient studies (<10) of any intervention to allow an assessment of the publication bias. All systematic reviews that performed quantitative synthesis were unable to get a higher score because of item 15, a critical domain on the AMSTAR-2 checklist, which consists of an adequate investigation of publication bias. This kind of study bias and its impact on the results of the review can only be performed with methodological rigor and enough studies on a determined pharmacological approach. The inter-rater agreement is shown in Table 2 ^11,12,13,14,15^. The domains were ordered according to the following sequence: Q1 = Search question (PICO); Q2 = Study planning (protocol); Q3 = Justification for the selection of the study design; Q4 = Search strategies; Q5 = Selection of peer studies; Q6 = Data extraction in pairs; Q7 = Report of excluded studies; Q8 = Characteristics of the studies included; Q9 = Risk assessment of bias; Q10 = Reporting of the sources of funding for the studies; Q11 = Appropriate statistical methods; Q12 = Assessment of the impact of the risk of bias in meta-analyses; Q13 = Risk of bias in interpretation and results; Q14 = Discussion and explanation of heterogeneity; Q15 = Investigation of publication bias; Q16 = Report of conflict of interest of the authors of the review.

**Table 2:** Risk of bias assessed through AMSTAR 2.

References	Q1	Q2	Q3	Q4	Q5	Q6	Q7	Q8	Q9*	Q9^†^	Q10	Q11*	Q11^†^	Q12	Q13	Q14	Q15	Q16	Quality ranking
Wang et al.^11^	Y	N	Y	Y	Y	Y	N	Y	Y	PY	N	N/A	N/A	N/A	Y	Y	N	Y	Critically low
Livingstone et al.^12^	Y	Y	Y	Y	Y	Y	Y	Y	Y	N/A	Y	Y	N/A	Y	Y	Y	N	Y	Low
Oliveira e Faria^14^	Y	N	Y	Y	Y	Y	N	Y	Y	N/A	N	N/A	N/A	N/A	Y	Y	N	Y	Critically low
Corniello et al.^15^	Y	Y	Y	Y	Y	Y	Y	Y	N/A	N	N	N/A	N/A	N/A	Y	Y	N	Y	Critically low
Islam et al.^13^	Y	Y	Y	Y	Y	Y	N	Y	Y	N/A	N	Y	N/A	N	Y	Y	N	Y	Critically low

Abbreviations: Y, Yes; N, No; PY, Partially yes; N/A, Non applicable.

Notes: *Randomized Controlled Trials;

^†^Non-randomized Studies of Interventions.

According to the evaluation by the GRADE approach, the quality of the evidence was low for a greater proportion of the pharmacological interventions.

The SR conducted by Islam et al.^13^ included studies that were all randomized controlled trials (RCTs), with a low risk of bias assessed for most areas, such as requested sequence generation, blinding and incomplete data. However, there was a risk of selective reporting bias in one of the studies, where some outcomes were not reported, which implies a reduction in the level of evidence. In the inconsistency domain, significant clinical heterogeneity between studies was demonstrated, including differences in treatment duration and participant characteristics. With only three studies included, it was not possible to perform a formal assessment of publication bias.

The Corniello et al. review^15^ mainly included case series and case reports, which generally have a higher risk of bias due to a lack of randomization and control. Clinical heterogeneity between studies was significant, with variations in patient age, seizure type, and interventions. Confidence intervals for therapeutic effects were wide, indicating uncertainty about the true efficacy of treatments, especially considering the small sample sizes in the included studies. The lack of a formal analysis of publication bias, combined with the limited number of studies, represents a potential source of publication bias.

The Cochrane review^12^ included RCTs, assessed using the Cochrane risk of bias tool. Most studies had a low risk of bias in the domains of sequence generation and blinding, but some had a risk of bias related to selective outcomes and incomplete data. A formal analysis of publication bias could not be performed due to the limited number of studies.

The Oliveira and Faria review^14^ included six RCTs, which were assessed for risk of bias using the Cochrane tool. Some studies were at risk of bias due to selective outcomes and incomplete data, particularly concerning memantine and vitamin E. Clinical heterogeneity was moderate, with variations in dose and duration of treatment, which may explain some of the differences in results. There was considerable imprecision in the confidence intervals, especially for the efficacy outcomes of memantine and intranasal insulin, indicating uncertainty about the true efficacy of the treatments. A formal analysis of publication bias could not be performed due to the limited number of included studies.

The Wang et al. review^11^ had wide confidence intervals, especially for efficacy endpoints in diseases such as Alzheimer’s and Down syndrome, indicating uncertainty about the true effects of L-carnitine. A formal analysis of publication bias was not conducted, but the limited number of studies may suggest a potential publication bias.

Furthermore, all studies were rated as high risk of imprecision and moderate level of inconsistency.

## DISCUSSION

DS has become increasingly diagnosed over the years, and, associated with this, advances in medicine have led to an increase in the life expectancy of those diagnosed. In this way, it has been observed that the early onset of AD, the dementia that most affects this population, is one of the main factors of morbidity with the aging of these individuals. This occurs due to an extra copy of the amyloid precursor protein (APP) gene on chromosome 21, which expresses the relevance of not only preventing, but also diagnosing early and treating people with DS appropriately in relation to AD^14,15^.

Among the drugs used in the treatment of AD and in reducing cognitive decline in people with DS, memantine, an NMDA antagonist, has been an option, which is already scientifically supported in people without DS, with efficacy in studies with rats with DS, but not yet well established in humans^13^. A systematic review and meta-analysis conducted by Islam et al.^13^ included three randomized, double-blind, placebo-controlled trial studies — which measured tolerability, efficacy and safety of memantine in patients with DS — and concluded that, statistically, there is no considerable therapeutic effect of memantine compared to placebo. However, in addition to having analyzed a very small number of studies and the individuals observed not having specified dementia, heterogeneity is observed in one of the clinical trials compared to the other two in terms of age range and duration of the therapeutic regimen, as well as the dose of memantine in the advance of therapy.

Such a difference in intervention can serve as an obstacle to a more accurate interpretation of the conclusions of the review. Thus, the need for more studies following similar evaluation patterns with the use of this therapy is evident. Livingstone et al.^12^, in turn, analyzed nine studies involving pharmacological interventions for cognitive decline in people with DS. The areas evaluated were: general functioning (including memory and thinking, speech, mood and behavior); cognitive functioning (including memory, keeping up with what is happening around you); adaptive behaviors (being able to perform daily tasks); or behavioral problems (such as irritability or aggression). In this aspect, within all the clinical trials analyzed by Livingstone et al.^12^, only the drug simvastatin (among donepezil, memantine, antioxidant mixture and acetyl-L-carnitine — in isolated studies with only one of these medications each) showed an advantageous result in assessments in relation to the improvement of memory when compared to placebo. Still, these were preliminary results, and the research with this conclusion is very small in number of participants. Despite this, such a result expresses that it is interesting to replicate studies with this statin, but with more individuals to confirm the suggested advantage.

The two clinical trials analyzing the efficacy of memantine by Livingstone et al.^12^, however, are the same ones that Oliveira and Faria^14^ and Islam et al.^13^ used in their reviews. It is noted that the time interval between the three reviews is seven and nine years between the first review and the second, and the first and the third, respectively, and this shows that the efficacy of memantine in people with DS has not been investigated more deeply in other research since 2012 — the date of publication of the two clinical trials studied in the three specified reviews. With this, it is understood that conducting research with memantine is still viable to better understand its potential therapeutic effect in this population. This is even more evident when it is observed that the physiology in metabolism, genetics and neurotransmitter activity in people with DS may favor an inadequate response to treatment with drugs such as memantine. Furthermore, there is the observation that the plasma concentration of this drug in people with AD and concomitant DS reaches levels lower than the therapeutic range in people without DS and with AD. Thus, studies with higher doses of memantine in people with DS are suggested to ensure whether there is viability of the use of this drug for prevention and treatment for cognitive decline in these individuals^13^.

Regarding the reduction of cognitive decline and, consequently, the morbidity of AD in people with DS, Corniello et al.^15^ propose, in their systematic review, routine neuropsychological evaluation as a screening strategy to identify the onset of AD and adequately initiate drug therapy to prevent late-onset myoclonic epilepsy in DS, which is related to AD in these individuals. In light of this situation, it can be understood that the use of medication in individuals with DS with a diagnosis of early cognitive decline, such as AD, can contribute to a better analysis of the dementia progression of these people.

Thus, a prospective study testing memantine in doses higher than those used for the treatment of AD in patients with DS in the initial period of cognitive decline seems to be an ideal scenario to study such a drug or even the previously mentioned simvastatin in search of treatment for these neuropsychiatric conditions associated with this population.

As for the use of acetyl L-carnitine, the studies analyzed do not necessarily address the population of interest in this review (people with DS); however, they suggest the importance of this substance for brain health, raising the possibility of the benefit of its use in the general population^11^. In view of this, there is not enough scientific evidence to support the use of L-carnitine to slow the progression of cognitive decline in people with DS, even when this decline is related to AD. Therefore, it is necessary to assess the risk-benefit of this substance for such individuals and investigate its relationship with the improvement or decline in the dementia progression of these individuals. It is suggested by us, therefore, that studies focusing on this aspect seek routine neuropsychological evaluations in these individuals, as proposed by Corniello et al.^15^, and evaluate acetyl L-carnitine in isolation, comparing it with placebo, so that more precise analyses can be performed.

Overall, there is a need for more research on the pharmacological treatments available for cognitive decline in individuals with DS. Despite the increasing diagnosis of AD in this population, studies indicate that most pharmacological therapies, including memantine and donepezil, have not shown significant improvements in cognitive function or related outcomes. The majority of the evidence has critically low quality, highlighting the challenges in establishing effective treatments.

Although some preliminary findings related to simvastatin have been observed, the limited number of studies and participants suggests that these results should be interpreted with caution. Furthermore, the lack of uniform reporting on the cognitive domains affected by pharmacological interventions complicates the assessment of their efficacy.

Given the physiological differences in individuals with DS, such as altered neurotransmitter activity and lower plasma concentrations of medications, there is an urgent need for more rigorous studies. Specifically, future research should focus on higher doses of memantine and explore the potential of simvastatin in larger cohorts to validate these preliminary findings.

At present, there is insufficient evidence to recommend any pharmacological therapies for cognitive decline in this population. Instead, emphasis should be placed on routine neuropsychological assessments to monitor cognitive changes over time. This proactive approach may help identify early signs of AD, allowing for the timely initiation of supportive care and non-pharmacological interventions that may enhance quality of life. Ultimately, comprehensive and methodologically sound research is crucial to identify effective treatment options and improve outcomes for individuals with DS.

## Data Availability

The data extracted from the included systematic reviews and used in the synthesis are available in the tables presented in this article.
