# ERRATUM: Current evidence on drug therapy for dementia in people with Down syndrome: an overview of systematic reviews

**DOI:** 10.1590/1980-5764-DN-2024-0241erratum

**Published:** 2026-03-06

The manuscript "Current evidence on drug therapy for dementia in people with Down syndrome: an overview of systematic reviews", DOI: https://doi.org/10.1590/1980-5764-DN-2024-0241, published in the Dement Neuropsychol 2025;19(Suppl 1):e20240241.

**Where it reads:**

Dement Neuropsychol 2025;19

**It should read:**

Dement Neuropsychol 2025;19(Suppl 1)

Editor-in-Chief: Sonia M. D. Brucki http://orcid.org/0000-0002-8303-6732

